# Financing Strategies to Facilitate Access to High-Cost Anticancer Drugs: A Systematic Review of the Literature

**DOI:** 10.34172/ijhpm.2021.138

**Published:** 2021-09-22

**Authors:** Chanthawat Patikorn, Suthira Taychakhoonavudh, Rungpetch Sakulbumrungsil, Dennis Ross-Degnan, Puree Anantachoti

**Affiliations:** ^1^Department of Social and Administrative Pharmacy, Faculty of Pharmaceutical Sciences, Chulalongkorn University, Bangkok, Thailand.; ^2^Department of Population Medicine, Harvard Medical School and Harvard Pilgrim Health Care Institute, Landmark Center, Boston, MA, USA.

**Keywords:** Expensive, Antineoplastic Drug, Reimbursement

## Abstract

**Background:** Each country manages access to anticancer drugs differently due to variations in the structure and financing of the health system, but a summary of the various strategies used is absent. This study aimed to review and summarize financing strategies implemented across countries to facilitate access to high-cost anticancer drugs.

**Methods:** We conducted a systematic review of articles referenced in PubMed, Embase, and Web of Science through May 12, 2021. Articles published in the English language from 2000 that describe strategies implemented in different countries to facilitate access to high-cost anticancer drugs were included. Letters, news articles, and proposed strategies were excluded. Quality assessment was not performed as we aimed to summarize the strategies. Data were analyzed by thematic analysis. A review protocol was registered at PROSPERO (CRD42018068616).

**Results:** The review included 204 studies from 176 countries. Three themes of financing strategies were identified: (1) Basic pharmaceutical reimbursement and pricing policies, (2) Alternative funding strategies specific to high-cost drugs, and (3) Financial assistance for individual patients. Access in most countries depends mainly on basic pharmaceutical reimbursement policies (165 of 176 countries). Apart from that, high-income countries (HICs) tended to use funding strategies targeting high-cost drugs (72% of HICs vs 0%-24% of the rest), such as managed entry agreements (MEAs) or dedicated funds for high-cost drugs. In contrast, lower-income countries tended to implement financial assistance programs for cancer patients as a tool to increase access (32% of HICs vs 62%-79% of the rest).

**Conclusion:** Many countries have implemented a combination of strategies to increase access to high-cost anticancer drugs. Most low- and middle-income countries utilized placement of anticancer drugs on a national list of essential medicines and patient assistance programs (PAPs) to facilitate access, while many HICs implemented a broader range of strategies.

## Background

 Cancer is a leading cause of death with 9.6 million deaths worldwide in 2018.^1^ Many effective therapies are available for cancer including surgery, radiation, and anticancer drugs. However, healthcare systems face challenges in providing access to anticancer drugs for patients who need them while controlling the overall cost of cancer care which has been increasing rapidly over the past two decades. Global expenditures on cancer therapies and supportive care drugs reached US$113 billion in 2016 and were expected to increase to more than US$137 billion by 2021.^2^

 Anticancer drugs are highly priced to reflect cost of lengthy research and development of both successful and unsuccessful drugs. Moreover, the use of anticancer drugs aimed for rare cancers are limited to relatively small number of patients. Because the life-threatening nature of cancer, patients are likely to express more willingness to pay for anticancer drugs, even though the benefits of prolongation of survival are limited.^3^ Therefore, the pharmaceutical companies could set price of anticancer drugs as high as the market could bear maximize profitable returns. It was found that the median annual price of anticancer drugs has been increasing from US$12 000 to more than US$120 000 over the past two decades.^2^ The recently developed anticancer drugs such as CAR T-cell therapy are even more expensive with treatment cost up to US$500 000 per year.^4^ Given the rising costs of anticancer drugs, payers are unlikely to provide unconditional reimbursement as a long-term solution. To stay within a pharmaceutical budget, reimbursements are often restricted to indications that provide demonstrated value for money. However, such restrictions can deny access to potentially useful therapy for patients in high need.

 Limited access to high-cost anticancer drugs is not only an issue in low- and middle-income countries^2,5,6^ but in high-income countries (HICs) as well.^7,8^ It was estimated in 2011 that only 15% of patients who lived in low- and middle-income countries in the Association of Southeast Asian Nations (ASEAN) had access to anticancer drugs compared with 55% of patients in Singapore, a HIC in ASEAN.^5^ Each country manages access to anticancer drugs differently due to variations in the structure and financing of the health system.

 Previous studies have mostly summarized strategies to facilitate access to high-cost anticancer drugs in a single country^7,9^ or a group of countries,^2,5,8,10^ but a summary of this disassembled evidence is absent. For a country to establish informed and effective strategies to deal with access to anticancer drugs, it is important to know the overall landscape of strategies implemented across the world, including not only HICs, but low- and middle-income countries as well. Therefore, the objective of this study was to review and summarize strategies implemented across countries at different income levels to facilitate access to high-cost anticancer drugs.

## Methods

###  Search Strategy and Selection Criteria

 This systematic review was conducted to summarize the financing strategies implemented in any countries to facilitate patients with cancer to access to anticancer drugs. This systematic review reported following the Preferred Reporting Items for Systematic reviews and Meta-Analyses (PRISMA) guideline^11^ (Supplementary file 1). A protocol of this review was registered at PROSPERO – CRD42018068616.

 The literature search was undertaken in May 12, 2021, using PubMed, Embase, and Web of Science. The search strategy used combinations of terms including “Policy,” “Program,” “Access,” “Cancer” and “Drugs” **(**Supplementary file 2). A search of the grey literature was not feasible to perform due to the lack of resources for non-English language.

 For purposes of this review, strategy was defined as any policies, policy instruments, programs, or activities established with the aim to facilitate access to anticancer drugs. We included a strategy when the focus of the article was anticancer drugs or if the authors stated that a general strategy had been implemented to facilitate access to anticancer drugs.

 The eligible studies are full-text articles published in the English language from 2000 that describe financing strategies implemented in different countries to facilitate access to high-cost anticancer drugs. Letters, news articles, and proposed strategies were excluded.

 After duplicates were removed, two reviewers (CP and ST) independently screened abstracts and titles for relevance. The full-text articles were independently selected by two reviewers (CP and ST).

###  Data Extraction

 One reviewer (CP) extracted data from selected studies in a data extraction form. The following data were extracted from the included studies: country, name of strategy, objective of strategy, description of strategy, initial year of implementation, initiators, and responsible organizations. The selected studies were then divided among the second (ST) and a third reviewer (PA) for cross-check of extracted data. Any discrepancies were resolved through a consensus discussion among reviewers.

###  Quality Assessment

 Quality and risk of bias assessment was not performed since this study aimed to describe and summarize the financing strategies.

###  Data Analysis

 The extracted data were analyzed using thematic analysis. One reviewer (CP) initially constructed coding framework by categorizing the extracted financing strategies based on how they aimed to facilitate access to anticancer drugs by targeting insurance coverage status, reimbursement, or price. Themes were developed from the coding process of the extracted data by adding new types of strategies and relevant sub-types, and refining the synthesized themes until saturation was reached when no more themes were identified. Subsequently, the synthesized themes were then discussed among researchers (RS, DRD, ST, and PA) until the themes were finalized.

 The identified countries were classified by the World Bank’s income levels to analyze the variation in the implementation of financing strategies across income levels.^12^ Results were summarized and presented by theme.

## Results

**Figure 1:**
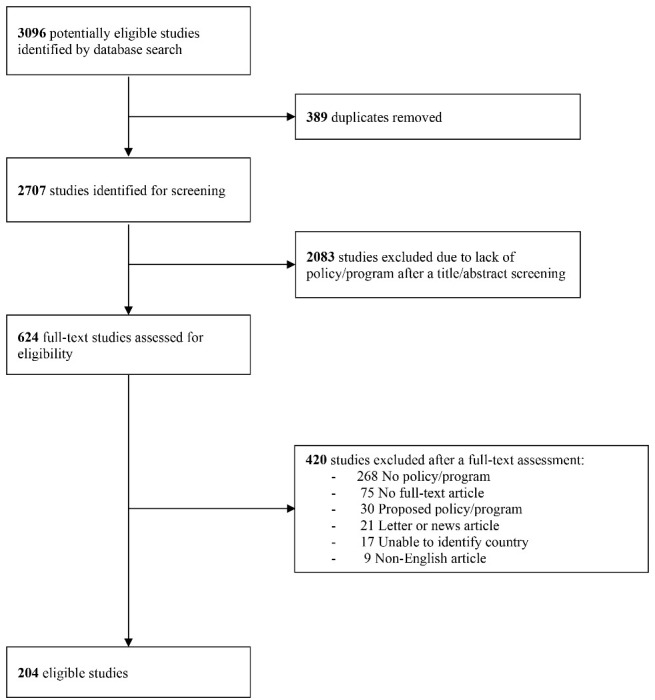
Study Selection Flow. Note: To ease presentation of the study selection process, the reasons for excluding studies after a full-text assessment were only described with the main reason.

 The search identified 3096 candidate studies of which 204 were included (Figure 1). A total of 176 countries was identified, comprising 53 HICs (30%), 50 upper middle-income countries (U-MICs) (28%), 47 lower middle-income countries (L-MICs) (27%), and 26 low-income countries (LICs) (15%). There were 172 studies describing strategies implemented in HICs, 56 studies in U-MICs, 25 studies in L-MICs, and 11 studies in LICs.

**Figure 2:**
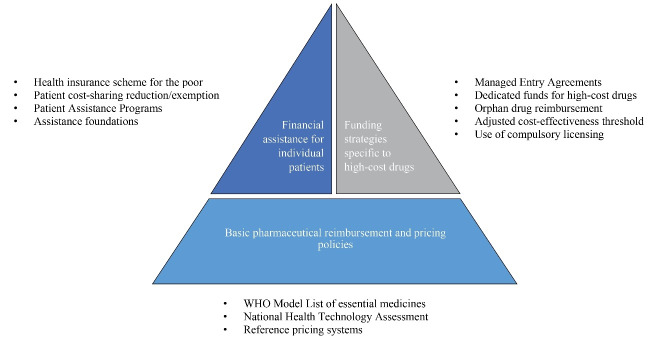
Themes and Sub-themes of Financing Strategies to Facilitate Access to High-Cost Anticancer Drugs. *Basic pharmaceutical reimbursement and pricing policies* included features of the basic reimbursement and pricing system in countries tailored to provide access to anticancer drugs to their population; *Funding strategies specific to high-cost drugs* included add-on strategies used specifically to provide access to high-cost drugs such as orphan or anticancer drugs when the basic pharmaceutical reimbursement and pricing policies were insufficient; *Financial assistance for individual patients* included add-on strategies intended to provide financial support for individual patients by either governmental or non-governmental organizations. Abbreviation: WHO, World Health Organization.

 We identified the 3 major themes with 12 sub-themes concerning strategies to facilitate patient access to high-cost anticancer drugs (Figure 2).Strategies in each country were summarized – (Supplementary file 3).The absence of strategies in countries indicated that they were not mentioned in the literature. Strategies were organized in the following categories:

1. *Basic pharmaceutical reimbursement and pricing policies* included features of the basic reimbursement and pricing system in countries tailored to provide access to anticancer drugs to their population;
2. *Funding strategies specific to high-cost drugs* included add-on strategies used specifically to provide access to high-cost drugs such as orphan or anticancer drugs when the basic pharmaceutical reimbursement and pricing policies are insufficient;
3. *Financial assistance for individual patients* included add-on strategies intended to provide financial support for cancer patients by either governmental or non-governmental organizations.

Among these, basic pharmaceutical reimbursement and pricing policies were the most commonly utilized policy (94% of 176 countries). Apart from the basic pharmaceutical reimbursement and pricing policies, HICs tended to use funding strategies for high-cost drugs (72% of HICs vs 0%-24% of the rest) while the rest implemented financial assistance programs for cancer patients as a tool to increase access (32% of HICs vs 62%-79% of the rest) as shown in Table 1 and Table 2.

**Table 1:** Themes of Financing Strategies by Country Income Level

**Income Level**	**Basic Pharmaceutical Reimbursement and Pricing Policies**		**Funding Strategies Specific to High-Cost Drugs**		**Financial Assistance for Individual Patients**	
	**No.**	**%**	**No.**	**%**	**No.**	**%**
High (n = 53)	49	92%	38	72%	17	32%
Upper middle (n = 50)	48	96%	12	24%	31	62%
Lower middle (n = 47)	44	94%	1	2%	37	79%
Low (n = 26)	24	92%	0	0%	17	65%
All (n = 176)	165	77%	51	29%	102	58%

**Table 2:** Sub-themes of Financing Strategies by Country Income Level

**Country**	**Basic Pharmaceutical Reimbursement and Pricing Policies**						**Funding Strategies Specific to High-Cost Drugs**										**Financial assistance for individual patients**							
	**WHO-EML**		**National Health Technology Assessment**		**Reference Pricing Systems**		**MEA**		**Dedicated Funds for High-Cost Drugs**		**Orphan Drug Reimbursement**		**Adjusted Cost-Effectiveness Threshold**		**Use of Compulsory Licensing**		**Health Insurance Scheme for the Poor**		**Patient Cost-Sharing Reduction/Exemption**		**PAP**		**Assistance Foundations**	
	**No.**	**%**	**No.**	**%**	**No.**	**%**	**No.**	**%**	**No.**	**%**	**No.**	**%**	**No.**	**%**	**No.**	**%**	**No.**	**%**	**No.**	**%**	**No.**	**%**	**No.**	**%**
High (n = 53)	24	45	40	75	26	49	31	58	18	34	16	30	6	11	1	2	1	2	4	8	13	25	2	4
Upper middle (n = 50)	45	90	20	40	5	10	7	14	7	14	3	6	0	0	1	2	3	6	2	4	30	60	0	0
Lower middle (n = 47)	43	91	4	9	2	4	0	0	0	0	0	0	0	0	1	2	1	2	0	0	37	79	0	0
Low (n = 26)	24	92	0	0	0	0	0	0	0	0	0	0	0	0	0	0	0	0	0	0	17	65	0	0
All (n = 176)	136	77	64	36	33	19	38	22	25	14	19	11	6	3	3	2	5	3	6	3	97	55	2	1

Abbreviations: PAPs, patient assistance programs; MEA, managed entry agreement; WHO-EML, World Health Organization Model Lists of Essential Medicines.

###  Basic Pharmaceutical Reimbursement and Pricing Policies

 Reimbursement and pricing policies are used by many countries to increase access to expensive medicines that are beyond most patients’ ability to pay.^13,14^ Reimbursement decisions are often made at the national level especially in countries where public health insurance is available. A national list of reimbursable medicines is also often referred to as a national drug formulary or a national list of essential medicines.^9,15,16^ In some countries, sub-national reimbursement schemes exist in which local, provincial or regional governments have reimbursement lists that are independent (eg, Canada)^17^ or that supplement the national reimbursement list (eg, China).^18^

 When local governments independently determine their own benefit scheme, significant geographic variations in access to anticancer drugs can occur, leading to a “postcode prescribing” phenomenon in which a patient’s access depends on where they live. For example, in Canada, only 7 out of 115 anticancer drugs were found to be available in all ten provinces.^19^ In Italy, although pricing and reimbursement decisions are made nationally, regional governments can charge different co-payments leading to price variations across the country.^10^

 Decisions to include anticancer drugs on national formularies are made with the guidance of several policy tools including the World Health Organization Model Lists of Essential Medicines (WHO-EML), health technology assessments (HTAs), and various price determination tools.

####  WHO Model Lists of Essential Medicines

 Since 1977, WHO has published the WHO-EML to guide countries in prioritizing access to essential medicines. In 2017, the WHO-EML included 40 anticancer drugs.^20^ WHO-EMLs are the most widely-used tools to increase access to anticancer drugs (77% of countries); however, their use is less common in HICs (45% of countries). U-MICs, L-MICs, and LICs (90%, 91%, and 92% of countries, respectively) used the WHO-EML as a guide to select medicines to include in their national drug formularies especially countries with low capacities and capabilities. However, the WHO-EML covers limited number of types of cancer with the median concordance between their national formularies in these countries and the WHO-EML was 42%.^15^

####  National Health Technology Assessment

 HTA is a multidisciplinary process for summarizing and evaluating information regarding efficacy, safety, cost-effectiveness (CE), and ethical and societal preferences for medical therapies and technologies to inform reimbursement decisions by payers. HTA was reported as a tool for reimbursement decisions in over half of HICs (75% of countries), with much lower use in U-MICs, L-MICs, and LICs (40%, 9%, and 0%, respectively).

 Each country has its own national HTA organization, process, and decision criteria.^21^ CE analyses which show value for money in the context of an individual health system are part of the HTA in some countries to efficiently select drugs for reimbursement and ensure affordability and sustainability of the health system. Countries may explicitly define a target CE threshold.^2,22^ Some countries, especially LICs and MICs, may comply with the WHO recommendation of three times the gross domestic product per capita as the CE threshold.^2^

 Since the HTA reviewing process can be lengthy, Canada and Denmark created special HTA pathway for anticancer drugs. In Canada, the pan-Canadian Oncology Drug Review did not publicly define an explicit CE threshold, which could result in decisions to reimburse high-cost anticancer drugs with relatively poor CE.^17^ In Denmark, the National Board of Health was once established in 2008 to separately review anticancer drugs for national reimbursement. The Danish Centre for Health Technology Assessment could rapidly review anticancer drugs within three months, compared with the normal process of one to two years for non-anticancer drugs. However, Danish Centre for Health Technology Assessment ceased to exist in 2012 when HTA in Denmark was shifted from the central to the regional level.^21^ These special HTA pathways facilitate patient access to high-cost anticancer drugs through prioritization of oncology before other therapeutic areas.

 HTA results, especially CE analyses and budget impact analyses, facilitate price negotiations and reimbursement price setting of a new product according to its added therapeutic value compared to existing treatments.^9,23^ Comparison criteria include, but are not limited to, quality-adjusted life years (QALYs) gained, degree of innovation, level of unmet need, lack of an effective alternative treatment, and burden of disease targeted by the product. This strategy is called “Value-based pricing” in Sweden and the United Kingdom.^24,25^ Prices of anticancer drugs may be set in reference to the country’s CE threshold. A system using an explicit or implicit CE threshold seeks to limit spending on drugs with low therapeutic value, and also incentivizes development of drugs with more added value. For example, in South Korea, there is a two-stage process for price negotiations, first an HTA-specific price negotiation process to lower price to a level consistent with the CE analysis followed by an obligatory price negotiation process with the South Korean payer.^23^ Although price negotiations could lower prices substantially, details in price negotiations in most countries were seldom disclosed.

####  Reference Pricing Systems

 The use of reference pricing was reported by HICs (49% of countries) with much lower use in U-MICs, L-MICs, and LICs (10%, 4%, and 0%, respectively). External reference pricing sets product prices based on a formula (eg, average, median, or other summary) using prices from several reference countries, while internal reference pricing sets a single reimbursement price for a group of drugs clustered by the mechanism of action, molecular similarity, or sometimes therapeutic effect. Some countries utilize external reference pricing to set the price of high-cost anticancer drugs, especially European countries where maximum prices were set based on the average price in other European countries which led to price transparency and information sharing across countries.^10,24,26^ Multinational pharmaceutical companies try to circumvent external reference pricing by limiting public information about their real prices (eg, by using confidential managed entry agreements, MEAs^27^) or delaying or avoiding product launches in countries with rigorous external reference pricing.^23^

 Internal reference pricing systems are also utilized in many European countries.^10,28^ For example, in Germany and the Netherlands, anticancer drugs with limited added benefits compared to existing therapies are priced at the level of similar drugs in a therapeutic class.^28,29^ This system allows greater patient access to several anticancer drugs within the same therapeutic class.

###  Funding Strategies Specific to High-Cost Drugs

 Included in this theme are specific strategies used to provide access to high-cost anticancer drugs and other high-cost treatments when the basic reimbursement and pricing policies proved to be insufficient. Most strategies are implemented by major public insurance payers using different tools and criteria. These funding strategies are not consistent across countries; some countries may use one strategy in conjunction with specific criteria while other countries may use the same strategy with different criteria.

####  Managed Entry Agreements

 MEAs are one of the most common funding strategies found in the literature. From 176 countries reviewed, 38 (22%) reported the use of MEAs to increase access to high-cost anticancer drugs. MEAs are a policy tool utilized when reimbursement decisions cannot be made due to uncertainties about clinical evidence, financial impacts or CE.^27^ MEAs are known as patient access schemes in the United Kingdom,^30^ managed access programs in Australia,^31^ and coverage with evidence development in the Netherlands.^29^ There are two main types of MEAs: performance-based MEAs that link drug reimbursement to a drug’s performance or patient outcomes and financial-based MEAs that indirectly lower drug prices through simple discounts, price-volume agreements, or rebates. Some MEAs utilize both.^27,30^

 MEAs facilitate access to high-cost anticancer drugs which would otherwise not be reimbursed at their offered prices by health system payers. The types and designs of MEAs vary from country to country. For example, most MEAs in Italy are performance-based with a refund for non-responders at the individual patient level while the majority of MEAs in the United Kingdom are financial-based with simple discounts.^27^

 MEAs have been increasingly used over time in HICs. One of the attractive attributes of MEAs is that significant price reductions can be achieved by payers while pharmaceutical companies can keep the negotiated net price undeclared. This hinders the effectiveness of external reference pricing since listed prices are not the real prices used.^27^

####  Dedicated Funds for High-Cost Drugs

 Some countries that have a societal preference for treatment of cancer over other diseases establish dedicated funds to subsidize access to high-cost anticancer drugs. Dedicated funds include special national budgets for high-cost drugs, additional payments for high-cost drugs, or special programs to provide access to drugs awaiting reimbursement decision.^9,10,25,32^ Dedicated funds for high-cost anticancer drugs were reported in 25 (14%) of countries, of which 18 are HICs.

 The well-known Cancer Drugs Fund (CDF) has been established in England in 2011. Drugs subsidized under the CDF are those receiving a negative recommendation from The National Institute for Health and Care Excellence (NICE) or those still in the reimbursement approval process.^25^ Due to rapidly increasing budgets, the CDF has been integrated into the NICE appraisal program since 2016. Drugs under the CDF will receive coverage with evidence development for two years with the chances of being delisted if further evidence shows no additional benefits or unresolved uncertainties.^33^ Therefore, dedicated funds require strong health system, technical capacity, and fund to appropriately decide which drugs to support and design monitoring system to generate evidence for further revision of the funds.

####  Orphan Drug Reimbursement

 Anticancer drugs that affect only a small proportion of the population or rare diseases are sometimes classified as orphan drugs, although the criteria vary from country to country. We found 16 HICs and 3 U-MICs reporting the use of orphan drug reimbursement. Some countries adjust CE thresholds to facilitate a reimbursement decision or provide full coverage without co-payments for orphan drugs.^32,34,35^

 Use of orphan drug status not only facilitates better access to medicine for patients with rare diseases but also incentivizes pharmaceutical companies to research and develop these drugs. Even though these criteria have not been specifically established for cancers, anticancer drugs would often be eligible.

####  Adjusted Cost-Effectiveness Threshold

 Because most innovative anticancer drugs are high-cost, they exceed most countries’ CE thresholds which would normally result in denial of reimbursement. However, six HICs (Australia, South Korea, the Netherlands, Slovakia, Sweden, and the United Kingdom) report use of higher CE thresholds as an add-on policy to the normal HTA process under certain circumstances, including expected number of patients, disease severity, medical need, lack of effective treatment, societal values, and expected impacts.^10,21,23,35,36^ These adjustments of CE thresholds are more common for anticancer drugs.

 In Australia, the “Rule of Rescue” allows reimbursement of cost-ineffective drugs treating severe and progressive diseases that affect a small number of patients when there is no existing alternative treatment.^21^ In the United Kingdom, the “End-of-life Criteria” were created specifically to fund life-prolonging drugs to treat diseases with short life expectancy by allowing incremental CE ratio beyond the usual CE threshold of £30 000. These criteria have led to a higher proportion of anticancer drugs approved for reimbursement.^35^

 Many countries allow higher CE thresholds depending on disease severity. However, the extent of CE threshold adjustments in most countries was not explicitly stated, with the exception of the Netherlands where the CE threshold can be adjusted between €20 000 and €80 000/QALY according to disease severity and medical need.^32^ Drugs reimbursed under the adjusted CE threshold are listed as innovative drugs and hospitals receive add-on payments for these high-cost drugs.^32^

####  Use of Compulsory Licensing

 The World Trade Organization Trade-Related Aspects of Intellectual Rights agreement allows any country with public urgent need to issue a compulsory license without consent from the patent holder to produce a generic drug. Also, the Doha Declaration allows countries without competency to produce local generic drug to import from other countries. Before issuing a compulsory license, the government may request a voluntary license from the pharmaceutical manufacturer.^5^ Compulsory licenses for anticancer drugs were reported only in Italy, India, and Thailand.^5,9,37^ In Thailand, the government issued compulsory licenses for three anticancer drugs (docetaxel, letrozole, and erlotinib) in 2008 to purchase generic drugs from India which resulted in a cost saving of more than US$140 million over 5 years.^9^ Compulsory licensing can significantly increase patient access to high-cost drugs, or serve as a negotiating tool to lower manufacturers’ drug prices, as seen in Colombia.^2^

###  Financial Assistance for Cancer Patients

 Some countries have developed strategies to help patients pay for their anticancer drugs out of pocket, either because they are not included in insurance reimbursement list or the patients are not enrolled in any health insurance scheme.

####  Health Insurance Scheme for the Poor

 We found five countries (the United States, China, Mexico, Russia, and India) where governments have established separate health insurance schemes to provide access to anticancer drugs for the poor or uninsured. Each scheme varied in its eligibility criteria, drug formulary, and benefits package. However, the schemes still failed to provide access to essential anticancer drugs and had insufficient coverage of the total costs of therapy.^18,38,39^

####  Patient Cost-Sharing Reduction/Exemption

 When high-cost anticancer drugs are not fully covered, patients face high cost-sharing. Treatments can be provided to eligible patients with co-payment reductions or exemptions. Such reductions/exemptions from cost-sharing were found in six countries including Croatia, France, the Netherlands, the United States, China, and Iran. However, most of these reductions/exemptions are not specific for anticancer drugs.^10,18^

####  Patient Assistance Programs

 Some pharmaceutical companies have developed patient assistance programs (PAPs) which donate anticancer drugs to patients who cannot afford them for low or no cost. PAPs are one of the most common ways that patients can access high-cost anticancer medicines when regular access through health insurance is limited. The programs and their regulation vary from country to country. PAPs were found in 97 countries (55% of 176 countries), comprising mainly low- and middle-income countries (84 of 176 countries).

 The most successful PAP is the Glivec/Gleevec International Patient Assistance Program (GIPAP) supported by Novartis. Since 2001, GIPAP had provided access to 63 000 patients in 93 countries who were otherwise unable to access imatinib.^40,41^

 Pharmaceutical companies use PAPs as one way to mask the real net price of their products in a country. They are also criticized as special marketing arrangements that offer indirect discounts, for example, the “buy 3 get 1 free” program reported by Sruamsiri et al in Thailand.^9^

####  Assistance Foundations

 As a last resort, patients may sometimes access affordable high-cost anticancer drugs through foundations or charities. The foundations were reported in Hong Kong and the United States. In Hong Kong, a charity called the Hong Kong Anti-Cancer Society has been established to assist patients who need financial support by giving cash subsidies and obtaining free drugs from pharmaceutical companies.^42^ In the United States, patient foundations, such as the Patient Access Network Foundation, provide financial support to Medicare patients. However, not every patient can obtain financial support, as each foundation has its own eligibility criteria, eligible drugs and diseases, and limited budget.^43,44^

## Discussion

 This systematic review is the first study to date which comprehensively summarizes financing strategies in 176 countries implemented to facilitate access to high-cost anticancer drugs during the last two decades. Although this review was focused on anticancer drugs, the financing strategies described in this study are frequently implemented for other high-cost drugs as well.

 For countries that face difficulty in ensuring access to high-cost anticancer drugs, this review can provide decision-makers with a broad view of the overall landscape of strategies to facilitate access to anticancer drugs in countries at different economic levels. However, which strategy will best serve a specific population will depend on the context of the health system in each country because there is no single strategy that could effectively facilitate access to anticancer drugs in every country. Likewise, different approaches are needed to improve access to anticancer drugs complementarily.

 Our systematic review found that a range of interacting financing strategies have been implemented to facilitate access to anticancer drugs, as shown in Figure 2. Basic pharmaceutical reimbursement and pricing policies serve as the fundamental first tier of strategies to provide access to high-cost anticancer drugs. HTA assists reimbursement decision making by evaluating the evidence and value for money of drugs under the country’s CE criteria. Countries use different pricing approaches to negotiate with manufacturers in order to encourage them to lower drug costs to an acceptable level. Most approaches are linked to listing specific drugs on the country’s reimbursement list, with many lower income countries using the WHO-EML as a reference.

 When basic pharmaceutical and pricing policies are not feasible or when they result in insufficient access to high-cost anticancer drugs, funding strategies specific to high-cost drugs and financial assistance for cancer patients may help to fill the gap in access. MEAs are beneficial primarily at an early stage of adding high-cost anticancer drugs to an insurance reimbursement list. In some circumstances, compulsory licenses can provide early access to generic anticancer drugs, which can result in substantial cost savings. Some PAPs provide financial assistance for patients who would otherwise not be able to afford needed anticancer drugs or when other strategies were not available to provide access. However, these alternative access strategies require some features of the basic pharmaceutical and pricing policies especially HTA which could be considered as the foundation to thoroughly and effectively develop informed strategies. For example, MEAs could not be efficiently implemented with appropriate designs if there is no HTA process in place to depict the uncertainties surrounding the decision to reimburse health technologies with could be worthily extended beyond costs per QALYs to include other values of anticancer drugs such as productivity, family spillovers, value of hope, and equity.^45^ Similarly, dedicated funds for high-cost drugs, orphan drug reimbursement, adjusted CE threshold, and use of compulsory licensing also require the existing HTA process.

 Our results indicate that each country implements various policies and programs according to unique health system objectives, in light of concerns about health system and patient affordability. HICs mainly implemented both basic pharmaceutical reimbursement and pricing policies and targeted funding strategies specific to anticancer drugs. In contrast, lower income economies mostly relied on financial assistance for cancer patients to supplement basic pharmaceutical reimbursement structures.

 It is unarguably that cancer affects patient’s and their family life tremendously both in terms of life lost and quality of life. Increasing access to anticancer that could save lives, thus, became an issue that many health policy makers are trying to achieve. It should be noted here that providing access to high-cost anticancer drugs will increase burden on the already constrained healthcare budget. This will raise concerns on equity, affordability, and sustainability of such access especially the opportunity costs of providing access to cancer instead of other diseases.

 Healthcare financing is one of the several core functions of the healthcare system. However, each healthcare system places different values on the treatment outcome of the patient. Even most financing strategies summarized in this study have an aim to achieve access to anticancer, the values of the treatment these financing strategies have tried to evaluate may be different. Therefore, to effectively adopt financing strategies from other countries to facilitate access to high-cost anticancer drugs, policy makers should thoroughly consider the potential benefits and challenges of financing strategy choices along with their country’s surrounding environment such as health insurance system, economy, infrastructure of health system, information system, capacity of human resources, and the existing policies. For instance, the priority of a country without national public insurance system might be to develop a national insurance for their population followed by supporting systems such as HTA before proceeding with alternative access pathways. Moreover, strong information system is necessary to support informed decisions to facilitate access to anticancer drugs not only selection process but also drug use monitoring to further refine the local treatment protocol in each country.

 Some limitations of this review and recommendation for future research are worth mentioning. One major limitation of this review is publication bias since we included only studies published in peer-reviewed journals in the English language and a search of the grey literature was not performed. Authors from HICs are much more likely to write on this topic for English-language journals. Limiting the review to published literature ensured a higher quality of the included studies, but constrained its ability to fully capture strategies that were not reported in the academic literature or evolving implementation details of the identified strategies. Some strategies might have been used only once or now ceased to exist. Some strategies might also have been replaced by the other strategies but were not explicitly reported in the published literature. For example, periodic price review of highly priced anticancer drugs may have been used in several countries to adjust the prices based on their actual budget impact but were not explicitly mentioned in the included articles. Moreover, the in-depth synthesis the social and economic environment surrounding the strategies in each country was not feasible given the data reported in the published literature. Therefore, a survey with key informants in each country would likely capture a more complete set of strategies to facilitate access to high-cost anticancer drugs. Furthermore, quality and risk of bias of the included studies was not performed. Thus, methodological issues and inherent risk of bias of the included studies such as reporting bias might be presented. Finally, while this review could describe strategies in different countries, the impacts of these strategies on access to anticancer drugs could not be fully evaluated, especially in light of the large differences in the health systems of the identified countries. For example, the need for supplementary funding strategies specific to anticancer drugs depends on the basic reimbursement framework of a country, ie, if every drug is fully reimbursed, there is no need for other measures to facilitate access. A future study with a narrower scope could provide more information about the necessities, impacts, and effectiveness of the strategies described in specific country contexts.

## Conclusion

 Many countries have implemented a combination of strategies reflecting national health system objectives and affordability in order to increase access to high-cost anticancer drugs for their populations. MICs and LICs tend to facilitate patient access by listing anticancer medicines on the national list of essential medicines and insurance reimbursement lists, supplemented by pharmaceutical industry-initiated PAPs. In contrast, many HICs have developed targeted funding strategies for high-cost anticancer drugs that seek to address clinical uncertainties and the relatively poor CE of these drugs.

## Ethical issues

 Not applicable.

## Competing interests

 Authors declare that they have no competing interests.

## Authors’ contributions

 Conception and design: CP, ST, RS, DRD, and PA. Acquisition of data: CP, ST, and PA. Analysis and interpretation of data: CP, ST, RS, DRD, and PA. Drafting of the manuscript: CP, ST, RS, DRD, and PA. Critical revision of the manuscript for important intellectual content: PA. Statistical analysis: CP. Obtaining funding: CP and PA. Administrative, technical, or material support: CP and PA. Supervision: PA.

## Data sharing

 The data that support the findings of this study are available from the corresponding author, PA, upon reasonable request.

## Funding

 This work was supported by the 90th Anniversary of Chulalongkorn University Scholarship Batch#41 (4/2018) Grant number 31. The funder had no role in any part of the work including design and conduct of the study, data collection, data management, data analysis and interpretation, preparation, review and approval of the manuscript.

## Supplementary files

Supplementary file 1. PRISMA Checklist.

Supplementary file 2. Full Search Strategy With Results.

Supplementary file 3. Strategies to Facilitate Access to High-Cost Anticancer Drugs by Country.
