# Supplementary material for: Financing Strategies to Facilitate Access to High-Cost Anticancer Drugs: A Systematic Review of the Literature
Source: Int J Health Policy Manag. 2021 Sep 22;11(9):1625–34. doi: 10.34172/ijhpm.2021.138 (PMC9808218; doi:10.34172/ijhpm.2021.138)
Supplement: Supplementary file 2 — Full Search Strategy With Results. [file ijhpm-11-1625-s002.pdf]

Article title: Financing Strategies to Facilitate Access to High-Cost Anticancer Drugs: A Systematic Review of the Literature

Journal name: International Journal of Health Policy and Management (IJHPM)

Authors' information: Chanthawat Patikorn[1], Suthira Taychakhoonavudh[1], Rungpetch Sakulbumrungsil[1], Dennis Ross-Degnan[2], Puree Anantachoti[1]*

[1]Department of Social and Administrative Pharmacy, Faculty of Pharmaceutical Sciences, Chulalongkorn University, Bangkok, Thailand.

[2]Department of Population Medicine, Harvard Medical School and Harvard Pilgrim Health Care Institute, Landmark Center, Boston, MA, USA.

(*Corresponding author: puree.a@chula.ac.th)

Supplementary file 2. Full Search Strategy With Results

Below are the full search strategy with results up to May 12, 2021 for three electronic databases including PubMed, EMBASE, and Web of Science. The search strategy used combinations of terms including "Policy", "Program", "Access", "Cancer" and "Drugs" with different searching technique specific to each databases. A total of 3096 publications was identified which comprised of 599 publications from PubMed, 1051 from EMBASE, and 1446 from Web of Science.

PubMed: May 12, 2021

	Search terms	*Hits*
#1	*Search (Policy[Mesh]) OR (Program OR Programs OR Programme OR Programmes)*	*1,669,986*
#2	*Search (Access OR Accessibility)*	*559,960*
#3	*Search Antineoplastic Agents[Mesh]*	*440,274*
#4	*Search (Neoplasm[Mesh]) AND Molecular Targeted Therapy[Mesh]*	*20,728*
#5	*Search (Antineoplastic Agents[Mesh]) OR ((Neoplasm[Mesh]) AND Molecular Targeted Therapy[Mesh])*	*450,731*
#6	*Search ((((Policy[Mesh]) OR (Program OR Programs OR Programme OR Programmes))) AND ((Access OR Accessibility))) AND ((Antineoplastic Agents[Mesh]) OR ((Neoplasm[Mesh]) AND Molecular Targeted Therapy[Mesh]))*	*672*
#7	*Search (((((Policy[Mesh]) OR (Program OR Programs OR Programme OR Programmes))) AND ((Access OR Accessibility))) AND ((Antineoplastic Agents[Mesh]) OR ((Neoplasm[Mesh]) AND Molecular Targeted Therapy[Mesh]))) Filters: Publication date from 2000/01/01; Humans; English*	*599*

EMBASE: May 12, 2021

	Search terms	*Hits*
#1	*policy OR policies OR program OR programs OR programme OR programmes*	*2,378,990*
#2	*access OR accessibility*	*744,666*
#3	*'antineoplastic agent' OR 'molecularly targeted therapy'*	*361,623*
#4	*#1 AND #2 AND #3*	*1,072*
#5	*#4 AND (2000:py OR 2001:py OR 2002:py OR 2003:py OR 2004:py OR 2005:py OR 2006:py OR 2007:py OR 2008:py OR 2009:py OR 2010:py OR 2011:py OR 2012:py OR 2013:py OR 2014:py OR 2015:py OR 2016:py OR 2017:py OR 2018:py OR 2019:py OR 2020:py OR 2021:py)*	*1,051*

Web of Science: May 12, 2021

	Search terms	*Hits*
#1	*TS=(Polic* OR Program*)*	*2,326,503*
#2	*TS=(Access OR Accessibility)*	*768,156*
#3	*TOPIC: (anticancer drugs)*	*73,695*
#4	*TS=(Cancer AND (Drugs OR Medications OR Pharmaceuticals))*	*294,905*
#5	*TS=(Antineoplastic agents OR Antineoplastic drugs OR Anticancer drugs OR Anti-cancer drugs)*	*99,791*
#6	*#5 OR #4*	*323,644*
#7	*TS=((Cancer OR Oncolog*) AND (Drugs OR Medications OR Pharmaceuticals))*	*301,203*
#8	*TS=(Drugs OR Medications OR Medicines OR Agents OR Pharmaceuticals)*	*3,470,818*
#9	*TS=(Cancer OR Oncolog* OR Hematolog* OR Haematolog* OR Neoplasm)*	*2,747,699*
#10	*#9 AND #8*	*501,722*
#11	*#10 AND #2 AND #1*	*1,540*
#12	*(#1 AND #2 AND #10) AND LANGUAGE: (English)*	*1,491*
#13	*(#1 AND #2 AND #10) AND LANGUAGE: (English); Timespan=2000-2021*	*1,446*
