# Supplementary material for: Financing Strategies to Facilitate Access to High-Cost Anticancer Drugs: A Systematic Review of the Literature
Source: Int J Health Policy Manag. 2021 Sep 22;11(9):1625–34. doi: 10.34172/ijhpm.2021.138 (PMC9808218; doi:10.34172/ijhpm.2021.138)
Supplement: Supplementary file 3 — Strategies to Facilitate Access to High-Cost Anticancer Drugs by Country. [file ijhpm-11-1625-s003.pdf]

Article title: Financing Strategies to Facilitate Access to High-Cost Anticancer Drugs: A Systematic Review of the Literature

Journal name: International Journal of Health Policy and Management (IJHPM)

Authors' information: Chanthawat Patikorn[1], Suthira Taychakhoonavudh[1], Rungpetch Sakulbumrungsil[1], Dennis Ross-Degnan[2], Puree Anantachoti[1]*

[1]Department of Social and Administrative Pharmacy, Faculty of Pharmaceutical Sciences, Chulalongkorn University, Bangkok, Thailand.

[2]Department of Population Medicine, Harvard Medical School and Harvard Pilgrim Health Care

Institute, Landmark Center, Boston, MA, USA.

(*Corresponding author: puree.a@chula.ac.th)

Supplementary file 3. Strategies to Facilitate Access to High-Cost Anticancer Drugs by Country

Country	Standard pharmaceutical reimbursement and pricing policies			Funding strategies specific to high-cost drugs					Financial assistance for individual patients			
	WHO List of essential medicines	National Health Technology Assessment	Reference pricing systems	Managed entry agreement	Dedicated funds for high-cost drugs	Orphan drug reimbursement	Adjusted cost-effectiveness threshold	Use of compulsory licensing	Health insurance scheme for the poor	Patient cost-sharing reduction/exemption	Patient Assistance Program	Assistance Foundations
**High-income countries**												
Antigua and Barbuda	/											
Australia		/	/	/	/	/	/				/	

**Country**	**Standard pharmaceutical reimbursement and pricing policies**			**Funding strategies specific to high-cost drugs**					**Financial assistance for individual patients**			
	**WHO List of essential medicines**	**National Health Technology Assessment**	**Reference pricing systems**	**Managed entry agreement**	**Dedicated funds for high-cost drugs**	**Orphan drug reimbursement**	**Adjusted cost-effectiveness threshold**	**Use of compulsory licensing**	**Health insurance scheme for the poor**	**Patient cost-sharing reduction/exemption**	**Patient Assistance Program**	**Assistance Foundations**
Austria		/		/								
Bahamas											/	
Bahrain	/											
Barbados	/										/	
Belgium		/	/	/	/							
Canada		/		/	/						/	
Chile	/	/			/						/	
Croatia	/	/	/	/		/				/		
Czech Republic	/	/	/	/								
Denmark		/	/		/							
Estonia	/	/	/	/								
Finland		/	/	/								
France		/	/	/	/	/				/		
Germany		/	/	/		/						
Greece		/			/							

Country	Standard pharmaceutical reimbursement and pricing policies			Funding strategies specific to high-cost drugs					Financial assistance for individual patients			
	**WHO List of essential medicines**	**National Health Technology Assessment**	**Reference pricing systems**	**Managed entry agreement**	**Dedicated funds for high-cost drugs**	**Orphan drug reimbursement**	**Adjusted cost-effectiveness threshold**	**Use of compulsory licensing**	**Health insurance scheme for the poor**	**Patient cost-sharing reduction/exemption**	**Patient Assistance Program**	**Assistance Foundations**
Hong Kong		/			/							/
Hungary		/	/	/		/						
Iceland		/	/									
Ireland		/	/									
Israel		/	/	/								
Italy		/	/	/	/			/				
South Korea	/	/	/	/		/	/				/	
Latvia	/	/	/	/	/	/						
Lithuania	/	/	/	/		/						
Luxembourg		/		/								
Malta	/	/		/								
Mauritius											/	
Monaco		/										
Nauru	/											
Netherlands		/	/	/	/	/	/			/		

Country	Standard pharmaceutical reimbursement and pricing policies			Funding strategies specific to high-cost drugs					Financial assistance for individual patients			
	**WHO List of essential medicines**	**National Health Technology Assessment**	**Reference pricing systems**	**Managed entry agreement**	**Dedicated funds for high-cost drugs**	**Orphan drug reimbursement**	**Adjusted cost-effectiveness threshold**	**Use of compulsory licensing**	**Health insurance scheme for the poor**	**Patient cost-sharing reduction/exemption**	**Patient Assistance Program**	**Assistance Foundations**
New Zealand		/		/							/	
Norway		/	/	/								
Oman	/											
Palau	/											
Panama											/	
Poland	/	/	/	/	/	/						
Portugal	/	/	/	/								
Romania	/	/	/	/		/						
Seychelles	/										/	
Singapore		/				/					/	
Slovakia	/	/	/		/	/	/					
Slovenia	/	/	/	/								
Spain		/	/	/								
St. Kitts and Nevis	/											
Sweden	/	/	/	/	/		/					

Country	Standard pharmaceutical reimbursement and pricing policies			Funding strategies specific to high-cost drugs					Financial assistance for individual patients			
	**WHO List of essential medicines**	**National Health Technology Assessment**	**Reference pricing systems**	**Managed entry agreement**	**Dedicated funds for high-cost drugs**	**Orphan drug reimbursement**	**Adjusted cost-effectiveness threshold**	**Use of compulsory licensing**	**Health insurance scheme for the poor**	**Patient cost-sharing reduction/exemption**	**Patient Assistance Program**	**Assistance Foundations**
Switzerland		/		/								
Taiwan		/		/	/	/						
Trinidad and Tobago	/											
United Kingdom		/		/	/	/	/					
United States				/	/	/			/	/	/	/
Uruguay	/	/			/						/	
**Upper middle-income countries**												
Albania			/								/	
Argentina	/	/									/	
Armenia	/										/	
Azerbaijan											/	
Belarus	/	/									/	
Belize	/											
Bosnia and Herzegovina	/	/		/	/							
Botswana	/										/	

**Country**	**Standard pharmaceutical reimbursement and pricing policies**			**Funding strategies specific to high-cost drugs**					**Financial assistance for individual patients**			
	**WHO List of essential medicines**	**National Health Technology Assessment**	**Reference pricing systems**	**Managed entry agreement**	**Dedicated funds for high-cost drugs**	**Orphan drug reimbursement**	**Adjusted cost-effectiveness threshold**	**Use of compulsory licensing**	**Health insurance scheme for the poor**	**Patient cost-sharing reduction/exemption**	**Patient Assistance Program**	**Assistance Foundations**
Brazil	/	/		/								
Bulgaria	/	/	/	/								
China	/	/		/					/	/	/	
Colombia	/	/									/	
Costa Rica	/										/	
Cuba	/											
Dominica	/											
Dominican Republic	/										/	
Ecuador	/	/									/	
Fiji	/										/	
Gabon	/										/	
Georgia	/										/	
Grenada	/											
Guatemala											/	
Guyana	/											

Country	Standard pharmaceutical reimbursement and pricing policies			Funding strategies specific to high-cost drugs					Financial assistance for individual patients			
	WHO List of essential medicines	National Health Technology Assessment	Reference pricing systems	Managed entry agreement	Dedicated funds for high-cost drugs	Orphan drug reimbursement	Adjusted cost-effectiveness threshold	Use of compulsory licensing	Health insurance scheme for the poor	Patient cost-sharing reduction/exemption	Patient Assistance Program	Assistance Foundations
Indonesia	/	/									/	
Iran	/	/								/		
Iraq	/											
Jamaica	/				/						/	
Jordan	/	/										
Kazakhstan		/			/	/					/	
Lebanon	/											
Macedonia	/	/										
Malaysia	/	/									/	
Maldives	/											
Marshall Islands	/											
Mexico	/	/	/		/				/		/	
Montenegro	/											
Namibia	/										/	
Paraguay	/										/	

Country	Standard pharmaceutical reimbursement and pricing policies			Funding strategies specific to high-cost drugs					Financial assistance for individual patients			
	WHO List of essential medicines	National Health Technology Assessment	Reference pricing systems	Managed entry agreement	Dedicated funds for high-cost drugs	Orphan drug reimbursement	Adjusted cost-effectiveness threshold	Use of compulsory licensing	Health insurance scheme for the poor	Patient cost-sharing reduction/exemption	Patient Assistance Program	Assistance Foundations
Peru	/										/	
Russia	/	/	/		/	/			/		/	
Serbia	/	/		/								
South Africa	/	/									/	
St. Lucia	/										/	
St. Vincent and the Grenadines	/											
Suriname	/										/	
Thailand	/	/		/	/			/			/	
Tonga	/											
Turkey		/	/	/		/					/	
Tuvalu	/											
Venezuela	/				/						/	
**Lower middle-income countries**												
Algeria	/											
Angola	/											

Country	Standard pharmaceutical reimbursement and pricing policies			Funding strategies specific to high-cost drugs					Financial assistance for individual patients			
	**WHO List of essential medicines**	**National Health Technology Assessment**	**Reference pricing systems**	**Managed entry agreement**	**Dedicated funds for high-cost drugs**	**Orphan drug reimbursement**	**Adjusted cost-effectiveness threshold**	**Use of compulsory licensing**	**Health insurance scheme for the poor**	**Patient cost-sharing reduction/exemption**	**Patient Assistance Program**	**Assistance Foundations**
Bangladesh	/										/	
Benin											/	
Bhutan	/										/	
Bolivia	/										/	
Cabo Verde	/										/	
Cambodia	/										/	
Cameroon	/										/	
Congo, Republic	/										/	
Côte d'Ivoire	/										/	
Djibouti	/											
Egypt	/											
El Salvador	/										/	
Ghana	/										/	/
Honduras	/										/	
India	/	/						/	/		/	

Country	Standard pharmaceutical reimbursement and pricing policies			Funding strategies specific to high-cost drugs					Financial assistance for individual patients			
	**WHO List of essential medicines**	**National Health Technology Assessment**	**Reference pricing systems**	**Managed entry agreement**	**Dedicated funds for high-cost drugs**	**Orphan drug reimbursement**	**Adjusted cost-effectiveness threshold**	**Use of compulsory licensing**	**Health insurance scheme for the poor**	**Patient cost-sharing reduction/exemption**	**Patient Assistance Program**	**Assistance Foundations**
Kenya	/										/	
Kyrgyzstan	/		/								/	
Kiribati	/											
Laos											/	
Lesotho	/										/	
Liberia	/											
Mauritania	/										/	
Moldova	/										/	
Mongolia	/										/	
Morocco	/										/	
Myanmar	/											
Nepal	/										/	
Nicaragua	/										/	
Nigeria	/										/	
Pakistan	/										/	

Country	Standard pharmaceutical reimbursement and pricing policies			Funding strategies specific to high-cost drugs					Financial assistance for individual patients			
	WHO List of essential medicines	National Health Technology Assessment	Reference pricing systems	Managed entry agreement	Dedicated funds for high-cost drugs	Orphan drug reimbursement	Adjusted cost-effectiveness threshold	Use of compulsory licensing	Health insurance scheme for the poor	Patient cost-sharing reduction/exemption	Patient Assistance Program	Assistance Foundations
Papua New Guinea	/										/	
Philippines	/										/	
Senegal	/										/	
Solomon Islands	/										/	
Sri Lanka	/										/	
Swaziland											/	
Tanzania	/										/	
Timor-Leste	/										/	
Tunisia	/											
Ukraine	/	/	/									
Uzbekistan		/									/	
Vanuatu	/											
Vietnam	/	/									/	
Zambia	/										/	
Zimbabwe	/										/	

Country	Standard pharmaceutical reimbursement and pricing policies			Funding strategies specific to high-cost drugs					Financial assistance for individual patients			
	WHO List of essential medicines	National Health Technology Assessment	Reference pricing systems	Managed entry agreement	Dedicated funds for high-cost drugs	Orphan drug reimbursement	Adjusted cost-effectiveness threshold	Use of compulsory licensing	Health insurance scheme for the poor	Patient cost-sharing reduction/exemption	Patient Assistance Program	Assistance Foundations
**Low-income countries**												
Afghanistan	/											
Burkina Faso	/										/	
Burundi	/											
Central African Republic	/										/	
Chad	/											
Congo, Democratic Republic	/										/	
Eritrea	/											
Ethiopia	/										/	
Gambia	/											
Guinea	/										/	
Haiti	/										/	
North Korea	/											
Madagascar	/										/	
Malawi	/										/	

**Country**	**Standard pharmaceutical reimbursement and pricing policies**			**Funding strategies specific to high-cost drugs**					**Financial assistance for individual patients**			
	**WHO List of essential medicines**	**National Health Technology Assessment**	**Reference pricing systems**	**Managed entry agreement**	**Dedicated funds for high-cost drugs**	**Orphan drug reimbursement**	**Adjusted cost-effectiveness threshold**	**Use of compulsory licensing**	**Health insurance scheme for the poor**	**Patient cost-sharing reduction/exemption**	**Patient Assistance Program**	**Assistance Foundations**
Mali	/										/	
Mozambique	/										/	
Niger											/	
Rwanda	/										/	
Sierra Leone											/	
Somalia	/											
Sudan	/										/	
Syrian Arab Republic	/											
Tajikistan	/										/	
Togo	/										/	
Uganda	/										/	
Yemen	/											
